# Associations Between Vegetarian Diet and Bioelectrical Impedance Parameters: Insights into Body Composition and Cellular Health in Young Adult Women

**DOI:** 10.3390/nu18020202

**Published:** 2026-01-08

**Authors:** Darina Falbová, Simona Sulis, Alexandra Hozáková, Petra Švábová, Radoslav Beňuš, Lenka Vorobeľová

**Affiliations:** Department of Anthropology, Faculty of Natural Sciences, Comenius University Bratislava, Ilkovičova 6, Mlynská Dolina, 842 15 Bratislava, Slovakia; sulis3@uniba.sk (S.S.); alexandra.hozakova@uniba.sk (A.H.); petra.svabova@uniba.sk (P.Š.); radoslav.benus@uniba.sk (R.B.)

**Keywords:** vegetarian diet, cellular health, body composition, young women

## Abstract

**Background:** Vegetarian diets are becoming increasingly popular among young adults. The aim of this study was to examine the association between adherence to a vegetarian diet and body composition parameters in young adult women. **Methods:** A sample of 647 young adult women, mainly university students from Slovakia, consisting of 66 vegetarians (22.02 ± 2.74 years old) and 581 omnivores (21.13 ± 2.20 years old) was analysed. Body composition was measured using a bioelectrical impedance analyser, the InBody 770. Information on participants’ diet was collected using a modified version of the WHO STEPS 2014 questionnaire. **Results:** Vegetarian women showed significantly lower phase angle (PhA) values compared with omnivores (5.06 ± 0.45 vs. 5.23 ± 0.48; *p* = 0.004). They also had a higher extracellular-to-total body water ratio (ECW/TBW; 0.382 ± 0.004 vs. 0.380 ± 0.005; *p* = 0.026). In multivariable linear regression adjusted for smoking status, physical activity, body mass index (BMI) and waist-to-hip ratio (WHR), vegetarian diet remained independently associated with lower PhA (β = −0.094; *p* = 0.011) and higher ECW/TBW (β = 0.085; *p* = 0.028). No significant indirect associations between a vegetarian diet and PhA or ECW/TBW through smoking status or physical activity were observed. **Conclusions:** In this study of young adult Slovak women, adherence to a vegetarian diet was independently associated with lower PhA and a higher ECW/TBW ratio. These findings indicate differences in BIA-derived indicators of cellular integrity and fluid distribution between vegetarian and omnivorous women, although causal relationships cannot be inferred.

## 1. Introduction

Vegetarian and plant-based dietary patterns have gained substantial popularity globally in recent years, with particularly high uptake observed among young adults. The motivations for these dietary choices are varied, including health awareness, ethical considerations, and environmental concerns. Young adults adhering to vegetarian diets are also more likely to prioritise organic, locally produced, ethically sourced, and non-genetically modified foods [1]. Additionally, approximately 35% of participants in a dietary intervention study expressed interest in adopting plant-based eating patterns [2].

Vegetarian dietary patterns have been consistently associated with a range of favourable health outcomes, including lower incidence of cardiovascular disease, type 2 diabetes, and hypertension [3,4]. These benefits are largely attributed to their nutritional profile, which typically features lower saturated fat intake and higher consumption of dietary fibre, whole grains, legumes, nuts, and soy protein [5]. In line with these nutritional characteristics, individuals following vegetarian diets often exhibit more favourable anthropometric profiles, including lower body mass index (BMI) and body weight compared with omnivorous counterparts [6,7]. However, despite extensive research on adiposity-related outcomes, considerably less is known about how vegetarian diets influence fluid balance and cellular health—key physiological markers that extend beyond traditional body composition measures. Understanding these associations may reveal additional pathways through which dietary patterns contribute to overall health.

At the same time, vegetarian diets may be associated with lower intake and biological availability of several critical nutrients predominantly found in animal-derived foods, including long-chain *n*-3 polyunsaturated fatty acids (e.g., docosahexaenoic acid, DHA), vitamin B12, zinc, and iron. These nutrients play essential roles in maintaining cell membrane structure, phospholipid composition, antioxidant defence, and intracellular–extracellular fluid regulation. Lower status of vitamin B12 and zinc has been linked to impaired redox balance and altered cellular metabolism, while reduced intake of long-chain *n*-3 fatty acids may influence membrane fluidity, inflammatory pathways, and cellular signalling. Such nutritional characteristics of vegetarian diets may therefore have implications not only for metabolic health but also for indicators of cellular integrity and hydration status [8,9].

Bioelectrical impedance analysis (BIA) represents a non-invasive and practical approach for assessing diet-related differences in body fluid distribution and cellular integrity. Resistance (R) and reactance (Xc) are fundamental BIA parameters used to derive indices such as phase angle (PhA), which reflects cellular health and membrane integrity [10]. Both Xc and PhA have shown predictive value for clinical outcomes, including protein–energy wasting and cardiovascular risk in haemodialysis patients [11,12]. Furthermore, the importance of fluid distribution is underscored by recent population research identifying the extracellular water–to–total body water ratio (ECW/TBW) as a significant health indicator with a non-linear association with all-cause mortality [13].

Although direct evidence linking vegetarian diets to PhA is limited, related findings suggest possible indirect pathways. Higher protein intake, including high-quality protein sources, has been identified as an independent predictor of PhA in athletes [14]. Similarly, studies among university students have reported strong associations between PhA and fat-free mass, TBW, muscle mass, and metabolic rate, with healthy lifestyle habits—such as regular physical activity and lower sedentary behaviour—showing positive relationships with PhA [15]. These findings suggest that both diet quality and lifestyle behaviours may jointly influence cellular health.

Despite these insights, research specifically examining the relationship between vegetarian diets and bioelectrical impedance parameters—particularly R, Xc, PhA, and ECW/TBW—remains scarce in young adult populations. This gap is particularly relevant given the growing prevalence of vegetarian diets among young adults and the potential implications for understanding cellular integrity, body composition, and fluid distribution.

Therefore, the present study aimed to determine whether adherence to a vegetarian diet is associated with bioelectrical impedance parameters (R, Xc, and PhA) and body fluid distribution (ECW/TBW) in young adult women, while accounting for lifestyle-related covariates such as physical activity and smoking status.

## 2. Materials and Methods

### 2.1. Participants

The research included a sample of 647 young adult women born between 1989 and 2005, mainly university students from Slovakia, comprising 66 vegetarians (22.02 ± 2.74 years old) and 581 omnivores (21.13 ± 2.20 years old). Participants were selected using a non-random method and voluntarily took part in assessments at the Biomedical Laboratory of the Department of Anthropology at Comenius University in Bratislava, Slovakia. Before the study began, each participant was assigned a unique identification number to ensure privacy. In accordance with the Declaration of Helsinki, written informed consent was obtained, and the study was approved by the Faculty of Natural Sciences Ethics Committee at Comenius University (approval number ECH19021). The study was designed as a population-based cross-sectional study. Apart from age (participants had to be between 18 and 30 years old) and sex, no exclusion criteria related to health status or lifestyle factors were applied.

### 2.2. Questionnaire

A standardised and validated questionnaire in Slovak was used to collect information on baseline characteristics. The questionnaire was an adapted version of the 2014 WHO expert questionnaire (STEPwise approach to surveillance—instrument version 3.2) [16]. The questions included in the study were as follows:

1. Are you a vegetarian? (yes, no);
  If the participant answered yes, a follow-up question was: How long have you been following a vegetarian diet? For this study, a participant was classified as a vegetarian if they had followed a vegetarian diet for at least six months.
2. Do you currently smoke any tobacco products, such as cigarettes, cigars or pipes? (yes, I am quitting smoking—I have not smoked for between one month and one year, I am quitting smoking—I have not smoked for at least one year, or no);
3. How often do you exercise or do physical activity? (>3 times per week, 1–2 days per week, <3 times monthly, or never).
4. How frequently do you consume alcohol? (daily, 5–6 days per week, 3–4 days per week, 1–2 days per week, 1–3 days per month, less than once a month, or never). Regular alcohol consumption was defined as alcohol intake on at least 1–2 days per week.
5. Do you regularly take hormonal contraceptives? (yes, no).

Information on alcohol consumption and use of hormonal contraceptives was included in supplementary analyses.

Physical activity, assessed using questions from Step 1 of the WHO STEPS instrument—which distinguishes between moderate- and vigorous-intensity activities—was then divided into three categories based on WHO recommendations for adults [17]. Tier 0 comprised participants who did not reach the minimum activity threshold and were limited mainly to light, incidental movement—such as walking to work—accumulating under 300 min of moderate activity or under 150 min of vigorous activity per week. Tier 1 included individuals who achieved 150–300 min of moderate exercise or 75–150 min of vigorous exercise weekly, as well as those exceeding these amounts but being active only once or twice per week. Tier 2 consisted of participants who consistently surpassed 300 min of moderate or 150 min of vigorous activity per week, typically engaging in exercise approximately three times weekly.

### 2.3. Anthropometric Analysis

All anthropometric assessments were conducted by trained anthropologists following standardised internationally recognised methods [18]. Height was measured with participants standing barefoot in an upright position and recorded to the nearest 0.5 cm using a Siber and Hegner anthropometer. Body weight was obtained to the nearest 0.1 kg using the built-in scale of the InBody 770 analyser. Body mass index (BMI) was calculated by dividing body weight (kg) by height squared (m^2^). Waist-to-hip ratio (WHR) was calculated as the ratio of waist circumference to hip circumference.

### 2.4. Body Composition Analysis

The InBody 770 analyser (Biospace Co., Seoul, Republic of Korea) was utilised to evaluate body composition. This instrument employs segmental multifrequency bioelectrical impedance analysis, which transmits low-level electrical currents through the body to measure tissue resistance. To ensure accurate results, the assessment was conducted under controlled conditions. Participants were instructed to abstain from physical activities for eight hours before the test, avoid consuming large amounts of food and water for three hours prior, stand barefoot on the pedal plate electrode, and hold the hand electrode at a 15° angle to prevent the arm from contacting the torso.

The instrument allows for the collection of data on body composition, such as phase angle (PhA), intracellular water (ICW; L), extracellular water (ECW; L), total body water (TBW; L), fat-free mass (FFM) and body cell mass (BCM; kg). In addition, we obtained the fat mass index (FMI), calculated as fat mass (kg) divided by height squared (m^2^): $FMI=FM\;(kg)/height^{2}\;(m^{2})$, the fat-free mass index (FFMI), calculated as fat-free mass (kg) divided by height squared (m^2^): $FFMI=FFM\;(kg)/height^{2}\;(m^{2})$. Lastly, total body Xc at 50 kHz was calculated, as the InBody device provides only segmental impedance (Z) and segmental Xc, but not total body Xc. The first step to obtain total body Xc is to calculate the segmental R. For each body segment—trunk, right leg, and right arm—the resistance is calculated using the following equation:

$$R\;seg=\sqrt{\left(\left({Z\;seg}^{2}\right)-({Xc\;seg}^{2})\right)}$$

The total body R is obtained by summing all the R segmental values:

R total body = R trunk + R right leg + R right arm

Finally, total body Xc can be calculated using the following equation:

$$Xc\;tot=R\;tot\times TAN\left(PhA\;tot\times \left(\frac{3.14}{180}\right)\right)$$

### 2.5. Statistical Analysis

All statistical analyses were conducted using SPSS version 26.0 (SPSS Inc., Chicago, IL, USA). Statistical significance was assessed using *p* values, with a threshold of *p* < 0.05. The one-sample Kolmogorov–Smirnov test was applied to assess the normality of the data distribution. Depending on the data distribution, mean and standard deviation (SD) were used to describe continuous variables. Student’s *t*-test and Mann–Whitney U test were used to compare body composition and somatic variables between vegetarians and omnivorous women. The selection of subsequent statistical tests depended on the results of the normality assessment. Differences between vegetarians and omnivorous women in categorical variables were tested using the Pearson Chi-square test in contingency tables. To examine the relationships between BIA parameters and specific predictor variables (smoking, physical activity, vegetarian diet, alcohol consumption, hormonal contraceptive use, BMI, WHR), a multiple forward regression analysis was conducted. Multicollinearity was assessed using variance inflation factors (VIFs), with all values below 2.0, indicating no relevant collinearity among predictors. A Generalised Linear Model (GLM) multiple mediation analysis was performed to examine direct and indirect associations between a vegetarian diet and body composition parameters (PhA and ECW/TBW), with BMI, WHR, smoking status, and physical activity included as parallel mediators.

## 3. Results

Table 1 presents the baseline characteristics, including demographic characteristics, lifestyle factors, and body composition parameters of the participants, divided into vegetarian women (*n* = 66; 10.20%) and omnivorous women (*n* = 581, 89.80%).

Vegetarian women had followed a vegetarian diet for an average of 5.93 ± 4.28 years. There were no statistically significant differences in smoking status between vegetarian and omnivorous women (*p* = 0.654). Physical activity also did not differ significantly between the groups (*p* = 0.232), with most participants in both groups being active only one to two times per week. Vegetarian and omnivorous women did not differ significantly in BMI or WHR. Similarly, no significant differences were observed between the groups in the prevalence of hormonal contraceptive use (*p* = 0.120). Likewise, the prevalence of regular alcohol consumption did not differ significantly between vegetarian and omnivorous women (*p* = 0.173).

Among body composition parameters, statistically significant differences were observed in PhA, with lower values in vegetarian women (5.06 ± 0.45 vs. 5.23 ± 0.48; *p* = 0.004), and ECW/TBW (0.382 ± 0.004 vs. 0.380 ± 0.005; *p* = 0.026), which was slightly higher in vegetarian women. No significant differences were found for other selected body composition parameters.

The forward method of linear regression analysis (Table 2) was used to examine the independent influence of selected factors (vegetarian diet, smoking, physical activity, BMI, and WHR) on the BIA parameters. The Durbin–Watson statistic indicated no autocorrelation in any of the models. The predictors, vegetarian diet, physical activity, BMI, and WHR had the greatest influence (*p* < 0.050) on PhA and ECW/TBW. For PhA, negative B coefficient values indicated that a vegetarian diet was associated with lower PhA values. Physical activity, BMI, and WHR had positive B coefficient values, indicating that higher physical activity levels and higher BMI and WHR values were linked to higher PhA values. For ECW/TBW, a vegetarian diet had a positive B coefficient, indicating that vegetarian women were associated with higher ECW/TBW values, while higher physical activity, BMI, and WHR values were each associated with lower ECW/TBW values.

Additional adjustment for alcohol consumption and hormonal contraceptive use did not alter the associations between vegetarian diet and bioelectrical impedance parameters.

**Figure 1:**
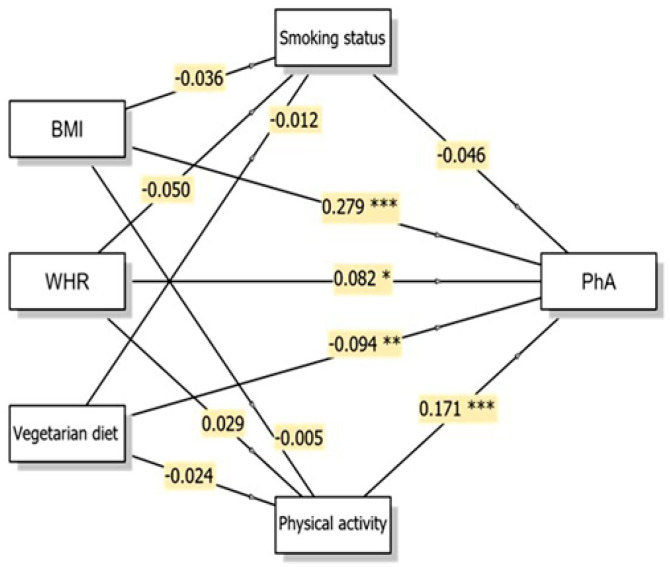
Conceptual diagram of the Generalised Linear Model (GLM) multiple mediation model showing direct and indirect pathways to phase angle with β coefficients. * *p* < 0.05; ** *p* < 0.01; *** *p* < 0.001. Abbreviations: BMI, body mass index; WHR, waist-to-hip ratio; PhA, phase angle.

Generalised linear mediation models were conducted to examine whether the associations of body composition indicators (BMI and WHR) and vegetarian diet with two key BIA measures (PhA and ECW/TBW) were mediated through smoking status and physical activity levels. PhA exhibited significant direct associations with all three independent variables (Figure 1). BMI demonstrated a strong positive association with PhA (β = 0.279, *p* < 0.001, 95% CI: 0.025–0.043). This positive relationship indicates that higher BMI was associated with greater cellular mass and potentially higher PhA values in this study sample. WHR also showed a significant positive direct association with PhA (β = 0.082, *p* = 0.030, 95% CI: 0.062–1.234), though the magnitude of this association was smaller than that of BMI.

Notably, none of the indirect pathways through smoking status (vegetarian diet → smoking status → PhA; β = 0.001, *p* = 0.767, 95% CI: −0.005–0.007) or physical activity (vegetarian diet → physical activity → PhA; β = −0.004, *p* = 0.548, 95% CI: −0.028–0.015) reached statistical significance for PhA. Thus, although physical activity showed a strong direct association with PhA (β = 0.171, *p* < 0.001), it did not significantly mediate the association between vegetarian diet and PhA. Overall, the associations between vegetarian diet and PhA remained after accounting for smoking status and physical activity, suggesting that these lifestyle factors do not substantially account for the observed relationship between vegetarian diet and PhA.

**Figure 2:**
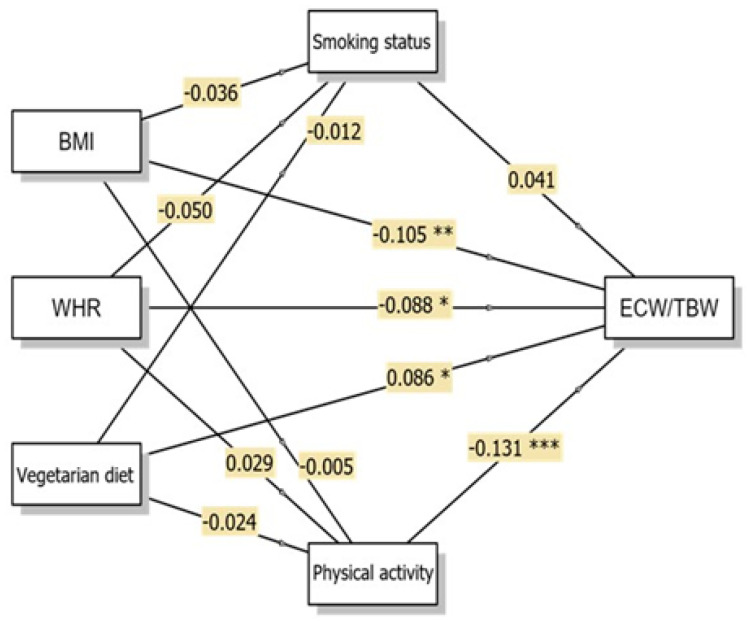
Conceptual diagram of the Generalised Linear Model (GLM) multiple mediation model showing direct and indirect pathways to ECW/TBW with β coefficients. * *p* < 0.05; ** *p* < 0.01; *** *p* < 0.001. Abbreviations: BMI, body mass index; WHR, waist-to-hip ratio; ECW/TBW, extracellular water/total body water.

The ECW/TBW ratio showed significant direct associations with all three independent variables, although the magnitudes of these associations were small (Figure 2). BMI demonstrated a small but significant negative direct association with ECW/TBW (β = −0.105, *p* = 0.008, 95% CI: −0.000–−0.000), indicating that higher BMI was associated with lower ECW/TBW ratios. This finding suggests that, within this sample, individuals with higher BMI tended to exhibit a lower proportion of extracellular water relative to total body water. WHR similarly showed a significant negative direct association with ECW/TBW (β = −0.088, *p* = 0.028, 95% CI: −0.013–−0.001). Consistent with the BMI results, higher WHR was associated with lower ECW/TBW ratios.

The vegetarian diet demonstrated a significant positive direct association with ECW/TBW (β = 0.086, *p* = 0.026, 95% CI: 0.000–0.003), indicating that vegetarian women exhibited relatively higher ECW/TBW values compared with omnivorous women. This pattern reflects differences in fluid distribution between dietary groups rather than implying specific physiological mechanisms. Physical activity emerged as a variable directly associated with ECW/TBW (β = −0.131, *p* < 0.001, 95% CI: −0.001–−0.000), with higher physical activity levels associated with lower ECW/TBW ratios.

However, none of the indirect pathways reached statistical significance, suggesting that smoking status (vegetarian diet → smoking status → ECW/TBW; β = −0.000, *p* = 0.770) and physical activity (vegetarian diet → physical activity → ECW/TBW; β = 0.003, *p* = 0.551) did not significantly mediate the association between vegetarian diet and ECW/TBW ratios in this study sample. Accordingly, the association between vegetarian diet and ECW/TBW remained after accounting for lifestyle factors such as smoking status and physical activity.

## 4. Discussion

The global distribution of vegetarianism shows marked regional variation, with the highest prevalence reported in Asia, where 19% of the population follows this practice. India, with almost 40% of its population being vegetarian, has the highest prevalence globally and contributes significantly to Asia’s figures [19,20]. In Africa and the Middle East, vegetarianism is observed in roughly 16% of the population, whereas in Central and South America, the prevalence is estimated at around 8%. The lowest proportions of vegetarian diets have been reported in North America (approximately 6%) and in Europe, where only about 5% of the population follows a vegetarian diet [19]. In our sample of young adult Slovak women, the prevalence of vegetarianism was 10.20%, which is higher than the general European average. The fact that our participants were university students may partly explain this higher proportion, as previous studies have shown that vegetarian diets are more common among individuals with higher education [21,22,23]. Similar results were observed in major Canadian cities, where a survey of youth and young adults aged 16–30 reported that 13.6% followed vegetarian dietary practices, including 6.6% vegetarians, 4.5% pescatarians, and 2.5% vegans [1]. Research in Germany indicated that 5.4% of adults adhered to vegetarian or vegan diets, with younger individuals showing a higher propensity for such dietary patterns [24].

Although we did not find statistically significant differences in BMI or WHR between vegetarian and omnivorous women in our study, numerous cross-sectional studies have reported more favourable adiposity-related outcomes among individuals adhering to vegetarian or plant-based diets [25,26,27]. Additionally, Karlsen et al. [28] found that BMI reduction was more pronounced in participants following diets such as Whole Food Plant-Based (WFPB), vegan, whole food, and low-carbohydrate diets for at least one year compared to those with shorter-term adherence, suggesting greater weight loss with longer dietary adherence. While the vegetarians in our sample reported long-term adherence to a vegetarian diet (mean duration 5.93 ± 4.28 years), both groups exhibited similar lifestyle characteristics strongly related to body weight regulation. Specifically, there were no differences in smoking status or physical activity levels, with most women in both groups exercising only once or twice per week. This homogeneity in lifestyle behaviours may have attenuated potential between-group differences in adiposity.

In the present study, adherence to a vegetarian diet was associated with body composition parameters, including PhA and ECW/TBW. However, these two markers reflect distinct, albeit related, physiological domains: PhA is considered an indicator of cellular quality and integrity, whereas the ECW/TBW ratio reflects fluid distribution and the extracellular compartment [29]. In clinical and population-based studies, higher PhA values have been associated with more favourable health outcomes, including reduced mortality risk and improved physical function [30]. Higher adherence to dietary patterns rich in high-quality fats, proteins, and antioxidants, such as the Mediterranean diet, has also been associated with higher PhA independent of age, sex, and BMI [14,31].

However, in the present study, vegetarian women exhibited lower PhA values, which is consistent with findings reported by Dawczynski et al. [32], who observed lower PhA and reduced cell quantity in men adhering to vegetarian dietary patterns.

While the underlying determinants of these differences cannot be established within the framework of the present study, the findings indicate that PhA may reflect qualitative characteristics of dietary patterns rather than overall adiposity alone.

Given that dietary intake was not quantitatively assessed, the observed association between vegetarian diet and lower PhA should be interpreted at a descriptive level, independent of specific nutritional mechanisms.

Vegetarian diets have been commonly associated with lower intake or bioavailability of certain nutrients, including long-chain *n*-3 polyunsaturated fatty acids, vitamin B12, zinc, and high-quality protein [8]. These factors represent biologically plausible explanatory considerations for differences in cellular-level BIA parameters; however, their role cannot be evaluated directly in the present analysis and requires confirmation in studies incorporating detailed dietary assessment.

Accordingly, references to nutritional pathways are presented as hypothesis-generating rather than causal interpretations.

At present, the clinical significance of small between-group differences in PhA and ECW/TBW in healthy young adult women remains unclear.

In our study, vegetarian women also exhibited significantly higher ECW/TBW values compared with omnivorous women. The ECW/TBW ratio is an important indicator of extracellular fluid expansion relative to total body water and is increasingly recognised as a marker of altered fluid distribution and impaired cellular hydration. Previous studies have associated elevated ECW/TBW with inflammation, fluid overload, and poorer clinical outcomes in various populations, including patients in intensive care settings [33]. Similarly, Wang et al. [13] reported that higher ECW/TBW may reflect subtle metabolic alterations, such as changes in sodium–water balance, vascular permeability, and low-grade inflammatory processes.

As no studies to date have directly examined ECW/TBW in vegetarian populations, the present findings should be considered novel but exploratory. Future studies incorporating detailed nutrient profiling, inflammatory markers, and objective measures of hydration status are therefore essential to clarify the physiological origins and potential clinical relevance of these differences.

### Limitations

This study has several limitations. First, its cross-sectional design precludes inference of causality. Second, the vegetarian classification did not consider diet quality, adherence, or specific subtypes (e.g., lacto-ovo, pescatarian, vegan), which may differently influence body composition parameters. This unmeasured heterogeneity within the vegetarian group may have contributed to within-group variability and potentially attenuated subtype-specific associations with BIA-derived parameters. Moreover, a detailed dietary assessment was not performed. The study focused on dietary pattern classification (vegetarian versus omnivorous diet) rather than quantitative evaluation of dietary intake. Therefore, food frequency questionnaires, 24-h dietary recalls, estimation of energy and nutrient intake, and comparisons with dietary reference values were not conducted. As a result, information on overall dietary quality, intake of key micronutrients (e.g., iron, vitamin B12, zinc, calcium), fatty acid composition, and supplement use was not available, which limits the ability to explore potential nutritional mechanisms underlying the observed associations. In addition, biological markers of nutritional and metabolic status, such as blood concentrations of vitamins, minerals, fatty acids, inflammatory markers, or indicators of oxidative stress, were not assessed, and total energy expenditure was not evaluated. While these factors could not be examined in the present study, they represent important targets for future research aimed at elucidating the physiological pathways linking vegetarian dietary patterns with cellular integrity and fluid distribution. Third, the sample consisted predominantly of young Slovak university women, restricting the generalisability of the findings to other age groups, socioeconomic backgrounds, geographic regions, and men. Fourth, this study relied on subjective self-reported assessments of environmental factors such as physical activity levels and smoking status, and important data may have been under-reported. Fourth, smoking status and physical activity were assessed using self-reported questionnaire data and categorised in a relatively broad manner, which may have limited sensitivity to detect more subtle associations or indirect pathways. This measurement imprecision may have contributed to the absence of significant mediation effects observed in the analyses. Despite these limitations, the study provides novel evidence linking vegetarian diets with BIA-derived markers of cellular integrity and fluid distribution in young women, underscoring the need for future research incorporating comprehensive dietary assessment, objective biomarkers of nutrient status and energy metabolism, and more diverse populations.

## 5. Conclusions

In this study of young adult Slovak women, adherence to a vegetarian diet was independently associated with lower PhA and a higher ECW/TBW ratio, even after adjustment for smoking, physical activity, BMI, and WHR. These findings indicate differences in BIA-derived indicators of cellular integrity and fluid distribution between vegetarian and omnivorous women. As research on bioelectrical impedance parameters in vegetarian populations remains limited, our results provide novel descriptive evidence of associations related to long-term vegetarian dietary patterns. Future studies including detailed dietary data, nutrient status, and inflammatory biomarkers are needed to clarify the underlying mechanisms and determine the clinical relevance of these findings.

## Figures and Tables

**Table 1:** Baseline characteristics of the participants.

	Total	Vegetarian Women	Omnivorous Women	*p*
Variables (*n*; %)	647	66 (10.20)	581 (89.80)	
**Demographic characteristics**				
Age (year)	21.22 ± 2.28	22.02 ± 2.74	21.13 ± 2.20	**0.009**
Smoking status (*n*; %)				
Current smoker	108 (16.69)	13 (19.70)	95 (16.35)	0.654
Past smoker (>1 month < 1 year)	22 (3.40)	1 (1.52)	21 (3.61)	
Past smoker (>1 year)	4 (0.62)	0 (0.00)	4 (0.69)	
Never	513 (79.29)	52 (78.79)	461 (79.35)	
Physical activity (*n*; %)				
Tier 0	172 (26.58)	16 (24.24)	156 (26.85)	0.232
Tier 1	312 (48.22)	38 (57.58)	274 (47.16)	
Tier 2	153 (25.19)	12 (18.18)	151 (25.99)	
Regular alcohol consumption				
Yes (*n*; %)	173 (26.74)	13 (19.70)	160 (27.54)	0.173
No (*n*; %)	474 (73.26)	53 (80.30)	421 (72.46)	
Hormonal contraceptive use				
Yes (*n*; %)	104 (16.07)	15 (22.73)	89 (15.32)	0.120
No (*n*; %)	543 (83.93)	51 (77.27)	492 (84.68)	
BMI (kg/m^2^)	22.06 ± 3.95	21.56 ± 2.88	22.12 ± 4.05	0.812
WHR	0.75 ± 0.06	0.76 ± 0.06	0.74 ± 0.06	0.062
**Body composition parameters**				
R (Ω)	687.87 ± 71.79	693.30 ± 76.81	687.25 ± 71.24	0.517
Xc (Ω)	62.48 ± 6.85	61.11 ± 5.68	62.64 ± 6.95	0.110
PhA (°)	5.21 ± 0.48	5.06 ± 0.45	5.23 ± 0.48	**0.004**
BCM (kg)	28.64 ± 3.44	28.29 ± 2.89	28.68 ± 3.49	0.429
FFM (kg)	43.88 ± 5.39	42.73 ± 6.34	44.01 ± 5.26	0.262
ICW (L)	20.02 ± 2.39	19.74 ± 2.00	20.06 ± 2.43	0.390
ECW (L)	12.29 ± 1.44	12.19 ± 1.26	12.30 ± 1.46	0.632
TBW (L)	32.39 ± 4.44	31.95 ± 3.27	32.44 ± 4.56	0.496
TBW/FFM	73.21 ± 0.17	73.25 ± 0.17	73.21 ± 0.17	0.067
ECW/TBW	0.381 ± 0.006	0.382 ± 0.004	0.380 ± 0.005	**0.026**
FMI (kg/m^2^)	6.19 ± 2.95	5.89 ± 2.04	6.23 ± 3.04	0.875
FFMI (kg/m^2^)	16.13 ± 7.92	18.58 ± 24.46	15.86 ± 1.44	0.365

Note: *p* values in bold are significantly less than 0.05. Abbreviations: *n*, number of participants; *p*, value of statistical significance; BMI, body mass index; WHR, waist to hip ratio; R, resistance, Xc, reactance; PhA, phase angle; BCM, body cell mass; FFM, fat-free mass; ICW, intracellular water; ECW, extracellular water; TBW, total body water; FMI, fat mass index; FFMI, fat-free mass index; Tier 0 comprised participants who did not meet the recommended physical activity levels (<300 min of moderate or <150 min of vigorous activity per week) and engaged primarily in incidental movement; Tier 1 included participants who met or slightly exceeded these thresholds but were active only one to two times per week; Tier 2 encompassed regularly active individuals who exceeded 300 min of moderate or 150 min of vigorous activity per week, typically training three times per week.

**Table 2:** Linear regression analysis of selected predictors with BIA parameters in the women’s study groups.

Dependents Variables	Predictors	Unstandardised β	Standardised β	95% CI for B	*p*	R^2^	Adjusted R^2^	Durbin-Watson	T
PhA	Vegetarian diet	−0.148	−0.094	−0.262–−0.034	**0.011**	0.139	0.134	2.107	0.992
	Physical activity	0.113	0.170	0.065–0.161	**<0.001**				0.999
	BMI	0.034	0.281	0.025–0.043	**<0.001**				0.930
	WHR	0.666	0.084	0.077–1.256	**0.027**				0.928
Excluded variable: smoking status									
ECW/TBW	Vegetarian diet	0.001	0.085	0.000–0.003	**0.028**	0.050	0.044	1.936	0.992
	Physical activity	−0.001	−0.130	−0.001–0.000	**0.001**				0.999
	BMI	0.000	−0.107	0.000–0.000	**0.008**				0.930
	WHR	−0.007	−0.090	−0.014–−0.001	**0.025**				0.928
Excluded variable: smoking status									

Note: *p* value in bold are significantly less than 0.05. Vegetarian diet (Vegetarian women vs. Omnivorous women). Abbreviations: B, unstandardised coefficient; Standardised β, Beta Coefficient; CI, confidence interval; *p*, value of statistical significance (regression analysis, stepwise method); R^2^, coefficient of determination; T, tolerance; PhA, phase angle; ECW, extracellular water; TBW, total body water; BMI, body mass index; WHR, waist to hip ratio.

## Data Availability

The original contributions presented in the study are included in the article, further inquiries can be directed to the corresponding author.

## Abbreviations

The following abbreviations are used in this manuscript:

BIA	Bioelectrical Impedance Analysis
PhA	Phase Angle
ECW/TBW	Extracellular Water/Total Body Water Ratio
ECW	Extracellular Water
ICW	Intracellular Water
TBW	Total Body Water
BMI	Body Mass Index
WHR	Waist-to-Hip Ratio
FFM	Fat-Free Mass
BCM	Body Cell Mass
FMI	Fat Mass Index
FFMI	Fat-Free Mass Index
R	Resistance
Xc	Reactance
Z	Impedance
GLM	Generalised Linear Model
SD	Standard Deviation
WFPB	Whole Food Plant-Based diet
WHO	World Health Organization
STEPS–WHO	Stepwise Approach to Surveillance
DHA	Docosahexaenoic acid
